# Brain-Derived Neurotrophic Factor Promotes Vasculature-Associated Migration of Neuronal Precursors toward the Ischemic Striatum

**DOI:** 10.1371/journal.pone.0055039

**Published:** 2013-01-29

**Authors:** Sofia Grade, Yuan C. Weng, Marina Snapyan, Jasna Kriz, João O. Malva, Armen Saghatelyan

**Affiliations:** 1 Cellular Neurobiology Unit, Insitut en Santé Mentale de Québec, Quebec City, Canada; 2 Center for Neuroscience and Cell Biology, University of Coimbra, Coimbra, Portugal; 3 Centre de Recherche du CHUL (CHUQ), Université Laval, Quebec City, Canada; 4 Department of Psychiatry and Neuroscience, Université Laval, Quebec City, Canada; 5 Center for Research on Environment, Genetics and Oncobiology (CIMAGO), Faculty of Medicine (polo 3), University of Coimbra, Coimbra, Portugal; Institut de la Vision, France

## Abstract

Stroke induces the recruitment of neuronal precursors from the subventricular zone (SVZ) into the ischemic striatum. In injured areas, de-routed neuroblasts use blood vessels as a physical scaffold to their migration, in a process that resembles the constitutive migration seen in the rostral migratory stream (RMS). The molecular mechanism underlying injury-induced vasculature-mediated migration of neuroblasts in the post-stroke striatum remains, however, elusive. Using adult mice we now demonstrate that endothelial cells in the ischemic striatum produce brain-derived neurotrophic factor (BDNF), a neurotrophin that promotes the vasculature-mediated migration of neuronal precursors in the RMS, and that recruited neuroblasts maintain expression of p75NTR, a low-affinity receptor for BDNF. Reactive astrocytes, which are widespread throughout the damaged area, ensheath blood vessels and express TrkB, a high-affinity receptor for BDNF. Despite the absence of BDNF mRNA, we observed strong BDNF immunolabeling in astrocytes, suggesting that these glial cells trap extracellular BDNF. Importantly, this pattern of expression is reminiscent of the adult RMS, where TrkB-expressing astrocytes bind and sequester vasculature-derived BDNF, leading to the entry of migrating cells into the stationary phase. Real-time imaging of cell migration in acute brain slices revealed a direct role for BDNF in promoting the migration of neuroblasts to ischemic areas. We also demonstrated that cells migrating in the ischemic striatum display higher exploratory behavior and longer stationary periods than cells migrating in the RMS. Our findings suggest that the mechanisms involved in the injury-induced vasculature-mediated migration of neuroblasts recapitulate, at least partially, those observed during constitutive migration in the RMS.

## Introduction

Adult stem cells in the subventricular zone (SVZ) of the lateral ventricle produce neuronal precursors that migrate toward the olfactory bulb (OB) via the rostral migratory stream (RMS). Interestingly, under certain conditions such as cortical or striatal strokes, neuronal precursor cells leave the SVZ and migrate toward ischemic areas [1]–[3]. In recent years, studies on post-stroke neurogenesis have revealed that recruited neuroblasts closely associate with blood vessels [4]–[6] and appear to travel along them [7], [8]. These data suggest that neuronal precursors require vasculature support for migration in post-stroke areas similar to the constitutive vasculature-mediated migration of neuroblasts in the RMS and OB [9]–[11]. However, the dynamics and molecular mechanisms driving the vasculature-mediated migration in post-stroke areas remain largely unexplored.

We previously pinpointed an important role for vasculature-derived brain-derived neurotrophic factor (BDNF) in promoting neuroblasts migration along the RMS via activation of p75NTR expressed by these migrating cells [10]. Stroke triggers the expression of BDNF in affected areas [12]–[14], and intravenous [15] or intraventricular [16] BDNF administration in animals subjected to phototrombotic ischemia leads to an increased number of SVZ-derived cells in injured tissues. It is, however, unclear whether BDNF directly affects the migration of neuroblasts in ischemic areas, and what the cellular sources of this trophic factor are. It has previously been shown that neurons in compromised areas transiently secrete BDNF [13], [14], [17]. In addition, BDNF immunolabeling has been observed in astrocytes, microglia, ependymal and endothelial cells at distinct times after injury [18]. However, since BDNF is a secreted protein that can be sequestered by other cell types, a detailed analysis of BDNF mRNA expression in post-stroke areas is required to determine its cellular source.

We studied the expression of BDNF and its receptors in the post-stroke striatum and explored BDNF involvement in the mechanisms of vasculature-mediated migration of neuronal precursors. Real-time imaging of cell migration revealed that BDNF promotes neuroblast displacement in the injured striatum along blood vessels that express this trophic factor. We demonstrated that injury-induced migration of neuroblasts shares similarities with the constitutive migration of neuronal precursors in the RMS with regard to (1) vasculature association, (2) expression of BDNF and its receptors, and (3) involvement of BDNF in the initiation of the migratory phase. Our results provide an insight into the mechanisms underlying injury-induced vasculature-mediated migration of neuronal precursors in ischemic areas.

## Materials and Methods

### Animals

We used adult, 2- to 3-month-old male C57BL/6 mice (Charles River, Wilmington, MA, USA). The experiments were approved by Université Laval Animal Care and Use Committee (permit number: 2010-173) and all efforts were made to minimize animal suffering and reduce the number of animals used. The mice were kept on a 12-h light/dark cycle at a constant temperature (22°C) and were given food and water *ad libitum*.

### Stereotaxic Injections

To label SVZ-derived cells, GFP-encoding lentiviruses or retroviruses were injected, 3 to 7 days before middle cerebral artery occlusion (MCAo), into the SVZ of adult C57BL/6 mice under ketamine/xylazine anesthesia (10 mg/1 mg per 10 g of body weight). The viruses were injected into both hemispheres into the dorsal and ventral sub-regions of the SVZ at the following coordinates (in mm): anterior-posterior 0.7; medial-lateral 1.2; dorsal-ventral 1.9 for dorsal SVZ; and anterior-posterior 0.9; medial-lateral 1.0; dorsal-ventral 2.7 for ventral SVZ, respectively. By combining two sites of injection in the SVZ, we were able to increase the number of infected cells and, as such, GFP-labeled de-routed neuroblasts. The mice that underwent stereotaxic injections received a single dose of ketoprofen (10 mg/kg) during the operation and were treated with the same analgesic during 3 days post-surgery. GFP-encoding viruses (150 nl, 1×10^6^–1×10^7^ TU/ml) were produced by the Platform for Cellular Imaging (http://www.neurophotonics.ca/en/platforms/molecular-tools-platform) according to the procedure described previously [10]. For real-time imaging in acute slices and to evaluate neuroblast/vasculature distances, the animals were sacrificed 3 to 4 weeks after the injections and 2 to 3 weeks post-MCAo.

### Middle Cerebral Artery Occlusion (MCAo)

Unilateral transient focal cerebral ischemia was induced by intraluminal filament occlusion of the left middle cerebral artery (MCA) for 1 h followed by reperfusion as previously described [19], [20]. Briefly, the animals were anesthetized with 2% isofluorane and kept at 37°C using a heating pad during the surgical procedure and reperfusion period. A midline neck incision was performed, and the internal carotid artery (ICA) was exposed by careful separation from the surrounding tissues under an operating microscope. A 12-mm-long, 6–0 silicon-coated monofilament suture was then introduced via the proximal left external carotid artery (ECA) into the ICA and then into the Circle of Willis to occlude the origin of the MCA. The wound was closed with a suture clip, and symptoms of stroke were monitored. After 1 h, the mouse was re-anesthetized to remove the filament and suture the skin. Mice were daily treated with saline solution during 1 week and analgesic during 3 days post-surgery. During all the post-operative period, weight loss and post-stroke symptoms were monitored. When gradual worsening of symptoms was observed, the mice were euthanatized to avoid unnecessary suffering of the animal.

### Tissue Processing and Vasculature Labeling with Dextran-texas Red

All the histological analyses were performed 1 and 2 weeks post-MCAo. The mice were deeply anesthetized and were perfused transcardially with saline solution (0.9% NaCl), followed by cold 4% paraformaldehyde (PFA). The brains were removed and kept in 4% PFA overnight at 4°C. They were then sectioned into 40-µm-thick sagittal slices using a vibratome (Leica, Concord, ON, Canada). The slices were processed as free-floating sections in phosphate-buffered saline (PBS). To assess how far neuroblasts migrated into the ischemic striatum we measured the nearest distance of Dcx-labeled neuroblasts from the SVZ or the RMS. To evaluate the association of neuroblasts with blood vessels, we either co-immunolabeled the fixed tissues for platelet endothelial cell adhesion molecule (PECAM) and Dcx or assessed retroviral-labeled GFP^+^ cells along dextran-Texas Red-filled blood vessels. For the latter case, 200 µl of high molecular weight dextran-Texas Red (70 000 kDa; Molecular Probes, Eugene, OR, USA) was injected into the heart of deeply anesthetized mice 2 min prior to removing the brains. All functional vessels in the brain can be visualized by Texas Red fluorescence using this approach. Dcx^+^ or GFP^+^ retroviral-labeled neuroblasts that had either the soma or leading process within 3 µm of PECAM or dextran-Texas Red-filled blood vessels were considered as vasculature-associated cells.

### *In situ* Hybridization

Antisense and sense RNA probes were generated from plasmids containing mouse BDNF (kindly provided by Dr. E. Castren, University of Helsinki, Finland) or the extracellular domain of mouse TrkB (kindly provided by Dr. Lino Tessarollo, National Institutes of Health, Bethesda, MD, USA). The riboprobes were labeled with digoxigenin (DIG) using DIG RNA labeling kits (Roche Diagnostics, Laval, QC, Canada) and were purified using ProbeQuant G-50 columns (GE Healthcare, Waukesha, WI, USA). Fixed sagittal brain slices (40-µm-thick) were treated with RIPA buffer (150 mM NaCl, 1% NP-40, 0.5% sodium deoxycholate, 0.1% SDS, and 1 mM EDTA in 50 mM Tris-HCl, pH 8.0). The slices were fixed in 4% PFA, washed in PBS, incubated in 0.1 M triethanolamine (TEA, pH 8.0), acetylated with 0.25% acetic anhydride in 0.1 M TEA, and washed again in PBS. The slices were then pre-incubated in hybridization solution (50% formamide, 5× saline sodium citrate (SSC), 5× Denhardt’s reagent, 500 µg/ml DNA, and 250 µg/ml of tRNA) for 1 h at 60°C. The riboprobe was added, and the slices were incubated overnight at 60°C. Afterwards, slices were washed in post-hybridization solution (50% formamide, 2× SSC, and 0.1% Tween-20), followed by buffer B1 (100 mM maleic acid, pH 7.5, 150 mM NaCl, and 0.1% Tween-20), and then buffer B2 (10% fetal bovine serum (FBS) in buffer B1). This was followed by an overnight incubation with anti-DIG antibody (1∶2000 dilution in buffer B2) at 4°C. After washing with buffer B1 and then buffer B3 (50 mM MgCl_2_, 100 mM NaCl, and 0.1% Tween-20 in 100 mM Tris HCl, pH 9.5), alkaline phosphatase activity was revealed using nitroblue-tetrazolium-chloride/5-bromo-4-chloro-indolylphosphate (NBT-BCIP; Promega, Madison, WI, USA). The slices were then washed in buffer B3 and mounted, or were fixed in 4% PFA for 30 min prior to the immunohistochemistry analysis.

### Immunohistochemistry

Free floating 40-µm-thick slices were washed in PBS, incubated in 0.2% Triton X-100 in PBS for 2 h, and then incubated overnight at 4°C (unless specified otherwise) with the primary antibody diluted in 4% bovine serum albumin (BSA) and 0.2% Triton X-100. The following antibodies were used: goat anti-Dcx (1∶1000; Santa Cruz Biotechnology, Santa Cruz, CA, USA); rat anti-mouse platelet endothelial cell adhesion molecule-1 (PECAM-1; 1∶100, three overnight incubations; BD Pharmingen, Mississauga, ON, Canada); rabbit (1∶1000; Dako, Burlington, ON, Canada) and mouse (1∶1000; Millipore, Billerica, MA, USA) anti-glial fibrillary acidic protein (GFAP); mouse anti-NeuN (1∶200; Millipore); rabbit anti-Iba1 (1∶400; Wako, Richmond, VA, USA); rabbit anti-Olig2 (1∶1000; Millipore); rabbit anti-brain-derived neurotrophic factor (BDNF; 1∶500, two overnight incubations; Santa Cruz Biotechnology, N-20); rabbit anti-TrkB (1∶1000, three overnight incubations; Millipore); rabbit anti-p75NTR (1∶500, three overnight incubations; Covance, Montreal, QC, Canada), and mouse anti-polysialic acid neural cell adhesion molecule (PSA-NCAM; 1∶1000; Millipore). The slices were then washed in PBS and were incubated with Alexa Fluor-conjugated secondary antibodies for 3 h (1∶1000 in PBS). Cell nuclei were counterstained with DAPI, and the sections were mounted in Dako fluorescent medium (Dako). An Olympus FV1000 (Richmond Hill, ON, Canada) confocal microscope was used to acquire and process images.

### PCR Analysis

To perform PCR analysis for BDNF, TrkB and p75NTR, ipsilateral and contralateral striata were dissected 7 days after MCAo and the total RNA was isolated using RNeasy Micro Kit (Qiagen), according to the manufacturer instructions. First strand cDNA synthesis reaction was performed with RevertAid H Minus First Strand cDNA Synthesis Kit (Fermentas Life science) with oligo(dT) primers. Obtained cDNA were amplified using following primers: for BDNF-Rs CGCCAGCCAATTCTCTTTTT and BDNF-Fw AAATTACCTGGATGCCGCAA; TrkB-Rs CATCCATCGGATGGGCAACAT and TrkB-Fw GGGGAAGGAGCCTTCGGG; for p75NTR-Rs CCCTACACAGAGATGCTCGGTTC and p75NTR-Fw CCAGCAGACCCACACACAGACTG; for GAPDH-Rs GTCCACCACCCTGTTGCTGTA and GAPDH-Fw TCCCATTCTTCCACCTTTGATG.

### Time-lapse Imaging

Two to three weeks post-MCAo, the mice were anesthetized, and the brains were quickly dissected in ice-cold sucrose-based artificial cerebral spinal fluid containing in mM: 210.3 sucrose, 3 KCl, 1.3 MgCl_2_, 2 CaCl_2_, 26 NaHCO_3_, 1.25 NaH_2_PO_4_, and 20 glucose (pH 7.4), bubbled with 95% O_2_–5% CO_2_. Sagittal sections (250 µm) were prepared using a vibratome (Leica) and were maintained at 32°C in artificial cerebral spinal fluid (ACSF) containing in mM: 125 NaCl, 26 NaHCO_3_, 3 KCl, 2 CaCl_2_, 1.3 MgCl_2_, 1.25 NaH_2_PO_4_, and 20 glucose (pH 7.4) bubbled with 95% O_2_–5% CO_2_. Individual slices were then placed in an imaging chamber mounted on a wide-field Olympus BX61WI upright microscope under continuous perfusion with ACSF (1 ml/min). The microscope was equipped with a CCD camera (CoolSnap HQ2, Photometrics, Tucson, AZ, USA) and a Lambda DG4 xenon light source (Sutter Instruments, Novato, CA, USA). The imaging chamber was connected to an automatic heating system (TC-344B, Harvard Apparatus, St. Laurent, QC, Canada) to maintain the ACSF at 31–33°C.

Time-lapse video images were captured using multiple *z*-stack acquisitions (6–16 *z*-sections, with 3–7 µm intervals) every 30 s for 1 h to track cell migration in the RMS or 2 h to track cell migration in the ischemic striatum. Fewer labeled cells were observed in the injured striatum, and they displayed fewer migratory events per hour. We thus tracked cell migration in the ischemic striatum using a longer acquisition time. The videos were processed and analyzed using Imaris software (Bitplane, South Windsor, CT, USA), which automatically tracks cells in 3D. A 0.045 µm/30 s threshold, which corresponded to the displacement between two subsequent acquisitions, was set to discriminate between the stationary and migratory phases. A careful visual inspection of cell migration revealed that migratory periods were consistently associated with values above 0.045 while stationary periods were associated with values below 0.045. Often the cell bodies of stationary cells wiggled slightly, when the cell screened the microenvironment with the leading process. The track lengths of migrating neuroblasts were automatically measured using Imaris software and were averaged for each video recording. These values were then averaged across all recordings and were compared. Cells that remained stationary for the duration of the recordings were not considered for analysis. To study the role of BDNF in injury-induced migration, we bath-applied BDNF (10 ng/ml; Peprotech) and TrkB-Fc (1 µg/ml; R&D Systems) and thereby modulate BDNF signaling. We used IgG-Fc (1 µg/ml; R&D Systems) as a control. For these experiments, time-lapse images were first acquired under control conditions for 1 h (ACSF) and then in the presence of the drug for another 1 h. Results are expressed as the percentage of the control value for each group obtained during the first hour of recording (ACSF). Cells tracked for less than 30 min before or after application of the pharmacological agent were not taken into consideration.

### Statistical Analysis

Data are expressed as means ± SEM. Values were analyzed using Student’s unpaired *t* test and were considered statistically significant at *p<0.05, **p<0.01, and ***p<0.001.

## Results

### Neuronal Migration in the Ischemic Striatum is Vasculature-dependent and Involves Astrocytes

**Figure 1:**
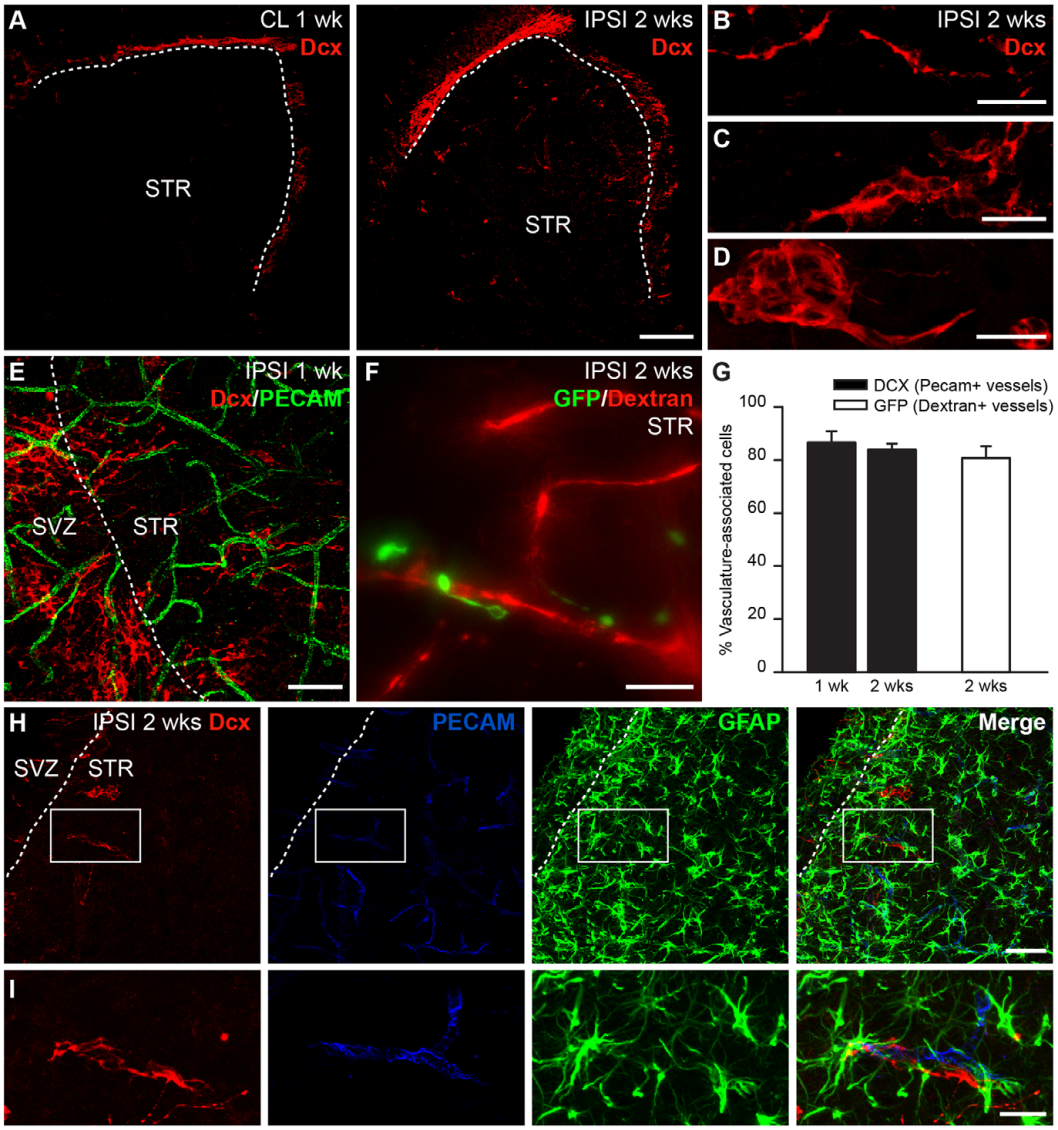
Migration of neuronal precursors in the ischemic striatum occurs along blood vessels and involves astrocytes. ***A***, Dcx immunoreactivity in the ipsilateral (right) and contralateral (left) striatum 2 weeks post-MCAo showing the presence of neuronal precursors in ischemic areas (right). In ischemic area, Dcx^+^ neuroblasts are found as individual cells (***B***) or are assembled in chains (***C***) or clusters (***D***). ***E,F***, Dcx^+^ neuroblasts (***E***) and GFP^+^ neuroblasts (***F***) are found in close proximity to PECAM (***E***) or dextran Texas Red-filled (***F***) blood vessels. ***G***, Vasculature-associated cells were quantified by counting the number of neuroblasts within 3 µm of blood vessels. ***H***, GFAP^+^ astrocytes were abundant and enveloped the vasculature that physically supported injury-induced migration. ***I***, High magnification image of the inset shown in (***H***). Scale bars: ***A***, 200 µm; ***B–D***,***F***,***I***, 20 µm; ***E***,***H***, 50 µm (SVZ: subventricular zone; STR: striatum; CL: contralateral; IPSI: ipsilateral; wk: week).

In the present study, we used a mouse model of MCAo, which has infarcts mainly in the striatum. To ascertain the efficiency of MCAo, the animals were tested, 24 hours after the operation, for hallmark neurological deficits associated with ischemia, such as circulating behavior, slight motor deficits on the contralateral front paw and reduced spontaneous activity. In addition, labeling with 2,3,5-triphenyltetrazolium chloride (TTC) 7 days after MCAo revealed pronounced infarct area in the ipsilateral striatum (data not shown). To evaluate the extent of neuronal migration from the SVZ to the infarcted striatum, we labeled for Dcx, a marker for newborn migrating neurons in the adult brain. Ischemia-induced neuroblasts recruitment toward the damaged striatum was triggered and maintained during the two first weeks following ischemia, which was in agreement with previous reports [21]. Small numbers of Dcx^+^ neuronal precursors had migrated to the striatum of the ipsilateral hemisphere within 1 week of MCAo (data not shown) while many more labeled cells that had migrated longer distances from the SVZ were observed 2 weeks following the injury (Fig. 1A, right). As reported previously [5], [8], [22], Dcx^+^ neuroblasts were observed as individual cells (Fig. 1B) or assembled in chains (Fig. 1C) or spherical clusters (Fig. 1D). Dcx^+^ cells, clusters or chains were found in the ischemic striatum at average distance of 128±9.7 µm from the SVZ or RMS (range from 7 to 406 µm, n = 82 cells/chains/clusters from 3 mice). In contrast, the corresponding contralateral striatum was devoid of Dcx immunoreactivity (Fig. 1A, left) and resembled that of the naïve brain. Co-immunolabeling for Dcx and PECAM revealed that the de-routed neuroblasts preferentially localized in the vicinity of striatal blood vessels (Fig. 1E,G). Indeed, 86.61±4.27% of Dcx^+^ cells (3529 cells analyzed, n = 5 mice) after 1 week and 83.95±2.24% of Dcx^+^ cells (2860 cells analyzed, n = 4 mice) after 2 weeks were located in the vicinity of PECAM^+^ blood vessels (Fig. 1E,G). This finding was consistent with previous reports [4]–[8] and suggested that neuroblasts use the vasculature as a physical substrate to support their migration to the post-stroke striatum. To ensure that the neuroblasts observed in the ischemic striatum had indeed migrated from the SVZ and had not been produced locally, we labeled the neuronal precursors in the SVZ by stereotaxic injection of GFP-encoding lenti- or retroviruses 3 to 7 days pre-MCAo. GFP^+^ neuroblasts were observed in the ipsilateral striatum and were preferentially associated with dextran-Texas Red-filled blood vessels 2 weeks post-MCAo (Fig. 1F,G; 80.75±4.44%, n = 119 cells, n = 17 mice).

Astrocytes play an important role in RMS neuroblasts migration by restraining chains within tubular glial structures [23], [24] and by affecting neuronal migration via releasable [25] and membrane-bound [26] factors. Astrocytes control extracellular levels of GABA to uphold neuroblast migration [27], modulate vasculature-mediated neuroblast migration [10], and regulate the formation of migration-promoting vasculature scaffolds [28]. We thus analyzed the arrangement of the astroglia in the ischemic striatum. Like the RMS, the striatal vessels used for post-stroke migration were ensheathed by astrocytes. We observed a massive astrogliosis in the injured striatum, where some of the reactive astrocytes were in close contact with neuroblasts and blood vessels (Fig. 1H,I). This cellular organization, which supports neuroblast migration toward the ischemic striatum, resembles the RMS cytoarchitecture, where constitutive migration of neuronal precursors toward the OB occurs [9], [10]. Since vasculature-derived BDNF has been shown to orchestrate neuroblasts migration in the RMS, we investigated the role of BDNF in injury-induced migration.

### Ischemia Induces BDNF Expression by Neurons and Endothelial Cells in the Striatum

**Figure 2:**
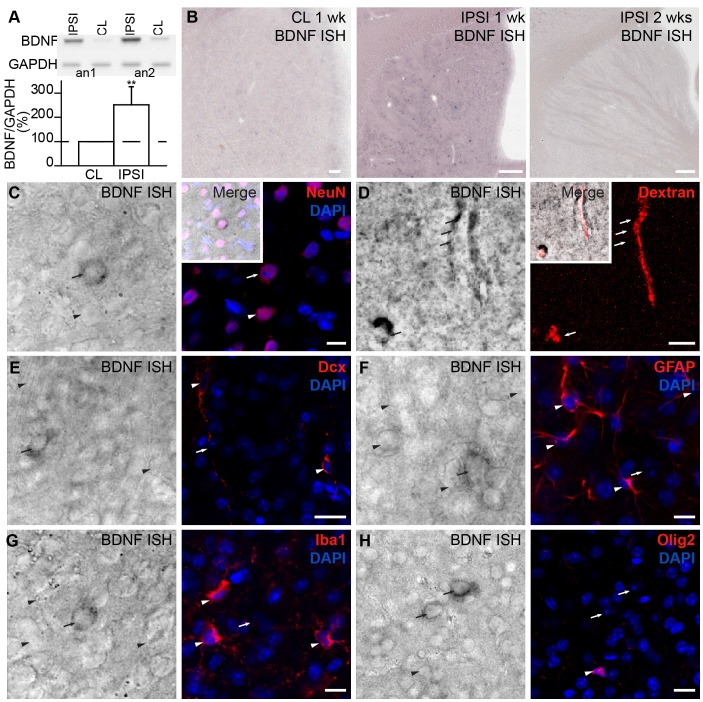
Ischemia induces BDNF mRNA expression by neuronal and endothelial cells in the ipsilateral striatum. ***A,*** PCR analysis of BDNF expression in the ischemic striatum 1 week after MCAo. Examples of contralateral (CL) and ipsilateral (IPSI) striata from 2 animals are shown. Low panel shows the quantification of BDNF expression in the ischemic striatum. ***B***, *In situ* hybridization revealed an upregulation of BDNF mRNA in rounded cells in the ipsilateral striatum 1 week post-ischemia (middle). Little or no BDNF mRNA was found in these cells 2 weeks post-ischemia (right). ***C–H***, High magnification images revealed that BDNF mRNA was expressed in NeuN^+^ striatal neurons (arrow) **(** ***C*** **)** and dextran-Texas Red-filled blood vessels **(** ***D*** **)**. ***E–H***, No BDNF mRNA was observed in Dcx^+^ neuroblasts (***E***), GFAP^+^ astrocytes (***F***), Iba1^+^ microglia (***G***), or Olig2^+^ oligodendrocytes (***H***) in the injured striatum. Arrowheads indicate cells negative for BDNF mRNA and arrows indicate cells positive for BDNF mRNA. Scale bars: ***B***, 200 µm; ***E***,***H***, 20 µm; ***C***,***D***,***F***,***G***, 10 µm (CL: contralateral; IPSI: ipsilateral; wk: week; ISH: *in situ* hybridization).

In the adult mice striatum, BDNF expression is extremely low and frequently undetectable [29], [30]. However, BDNF levels are transiently increased following striatal injuries [13], [31], [32]. To quantify the level of BDNF expression 1 week after MCAo we performed PCR analysis for BDNF in the contralateral and ipsilateral striata. In agreement with previous reports [13], [31], [32], at 1 week post-MCAo, BDNF mRNA expression in the ipsilateral striatum had increased to 251.0±75.9% (*p*<0.01; n = 4 mice) (Fig. 2A). To study the cellular pattern of BDNF expression, we first performed an *in situ* hybridization to detect BDNF mRNA. BDNF mRNA levels in the contralateral striatum at 1 or 2 weeks post-MCAo (Fig. 2B, left) were similar to the naïve brain (data not shown), with no or few positive cells per slice. We did, however, detect an upregulation of BDNF mRNA expression in dispersed round-shaped cells (Fig. 2B, middle) in the ischemic striatum 1 week post-MCAo. At 2 weeks post-MCAo, BDNF mRNA expression in these cells had dropped to contralateral levels (Fig. 2B, right). No signal with sense BDNF riboprobe was detected in the contralateral and ipsilateral striata (Fig. S1). *In situ* hybridization combined with immunohistochemistry for cell type-specific markers revealed that the round-shaped cells are NeuN^+^ neurons (Fig. 2C). Interestingly, in the ischemic striatum, BDNF mRNA was also detected in dextran-Texas Red-filled blood vessels (Fig. 2D). No BDNF mRNA was observed in blood vessels in the contralateral or naïve striatum (data not shown). While BDNF expression in neurons was upregulated 1 week post-MCAo and subsequently dropped to control levels after 2 weeks, BDNF expression in striatal blood vessels persisted 2 weeks after the injury. No BDNF mRNA was detected in other cells types in the ischemic striatum, including Dcx^+^ neuroblasts (Fig. 2E), GFAP^+^ astrocytes (Fig. 2F), Iba1^+^ microglia (Fig. 2G), and Olig2^+^ oligodendrocytes (Fig. 2H).

**Figure 3:**
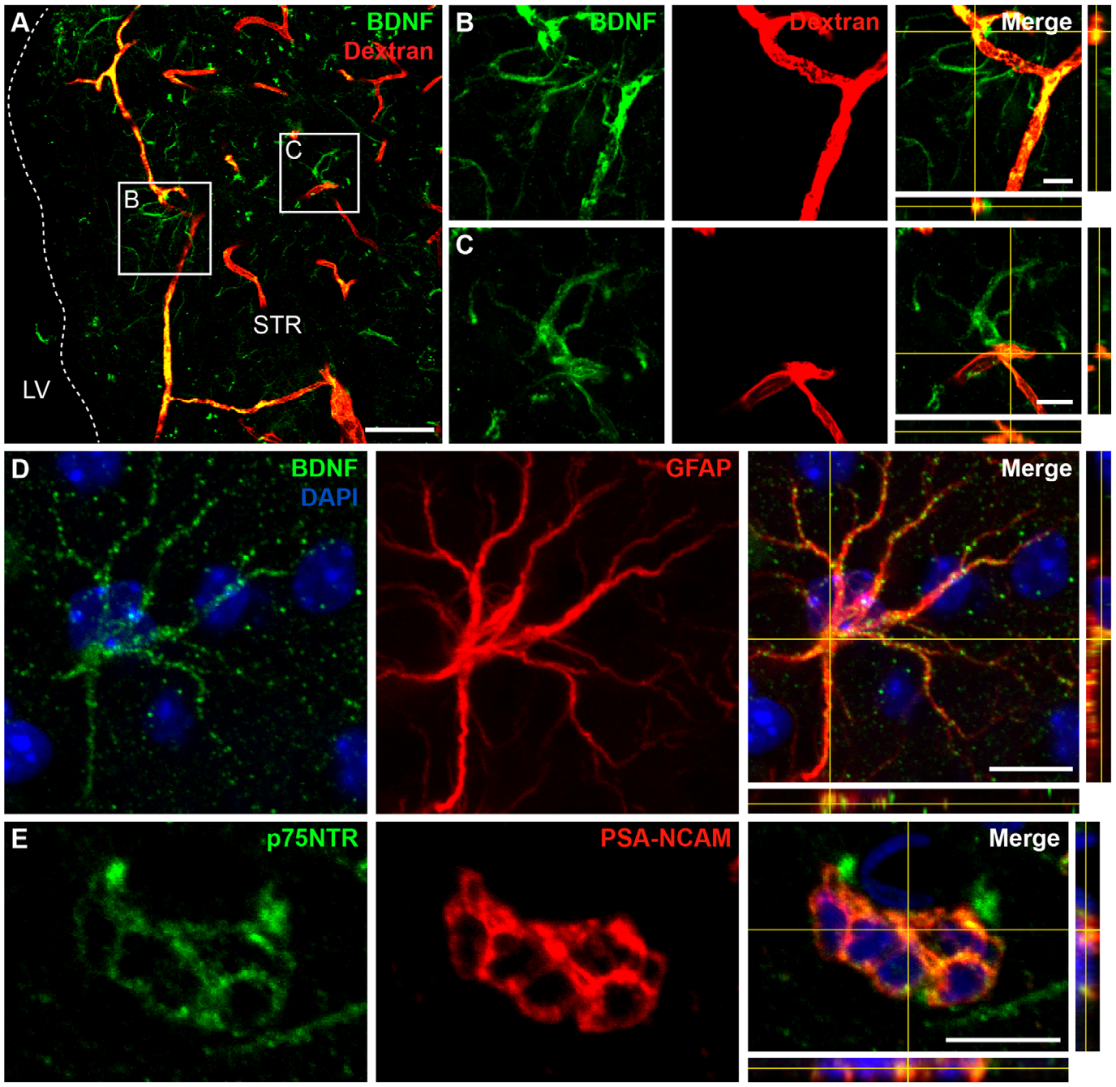
Expression of BDNF and p75NTR in the post-stroke striatum. *A* , BDNF immunolabeling was observed in dextran-Texas Red-filled blood vessels and in astrocytic-like cells. ***B***,***C***, High magnification images from insets depicted in (***A***) showing astrocytic-like cells in close proximity to blood vessels. Note that the blood vessels and astrocytes are immunopositive for BDNF. ***D***, Co-immunolabeling for BDNF and GFAP revealed the presence of BDNF protein in astrocytes. ***E***, PSA-NCAM^+^ migrating neuroblasts maintained p75NTR expression when de-routed to the injured striatal matrix. The pattern of expression shown in these panels was observed 1 and 2 weeks post-MCAo. Scale bars: ***A***, 50 µm; ***B–E***, 10 µm.

Having shown that BDNF mRNA is expressed, we investigated the expression of BDNF protein. Surprisingly, we detected BDNF immunopositive signals not only in dextran-Texas Red-filled blood vessels (Fig. 3A) and a few neurons (data not shown), but also in astrocytic-like cells, some of which were closely associated with the dextran-Texas Red-filled blood vessels (Fig. 3A–D). Co-labeling for GFAP and BDNF confirmed that astrocytes in the ischemic striatum are immunopositive for BDNF (Fig. 3D). These results were observed 1 and 2 weeks post-injury. Given that no BDNF mRNA was detected in astrocytes (Fig. 2F), this finding led us to hypothesize that BDNF secreted by endothelial cells and/or neurons following an injury may be trapped by neighboring astrocytes. Since neuronal BDNF mRNA expression is drastically reduced 2 weeks post-injury, it is conceivable that endothelial cells that maintain BDNF expression at 2 weeks are the main cellular source for this secreted trophic factor. Interestingly, we previously demonstrated that astrocytes in the RMS bind endothelial BDNF via cell-surface TrkB receptors and thus modulate its availability for p75NTR-expressing migrating neuroblasts [10]. This led us to analyze the patterns of TrkB and p75NTR expression in the ischemic striatum.

### Migrating Neuroblasts in the Damaged Striatum Express p75NTR, While Reactive Astrocytes Express TrkB Receptor

We observed p75NTR immunolabeling in PSA-NCAM^+^ neuroblasts that had de-routed to the site of injury (Fig. 3E). We also detected p75NTR immunolabeling in astrocytes and some neurons of the ipsilateral striatum (data not shown). These data were consistent with previous reports showing that p75NTR is upregulated in reactive astrocytes in the ischemic hippocampus [33], [34] and in cholinergic neurons in the ischemic striatum [35]. However, no significant changes in p75NTR mRNA were detected in the ipsilateral striatum by PCR analysis (104.8±13.3% of change, n = 4 mice). This is probably due to low number of de-routed neuroblasts as compared to the amount of striatal cells.

**Figure 4:**
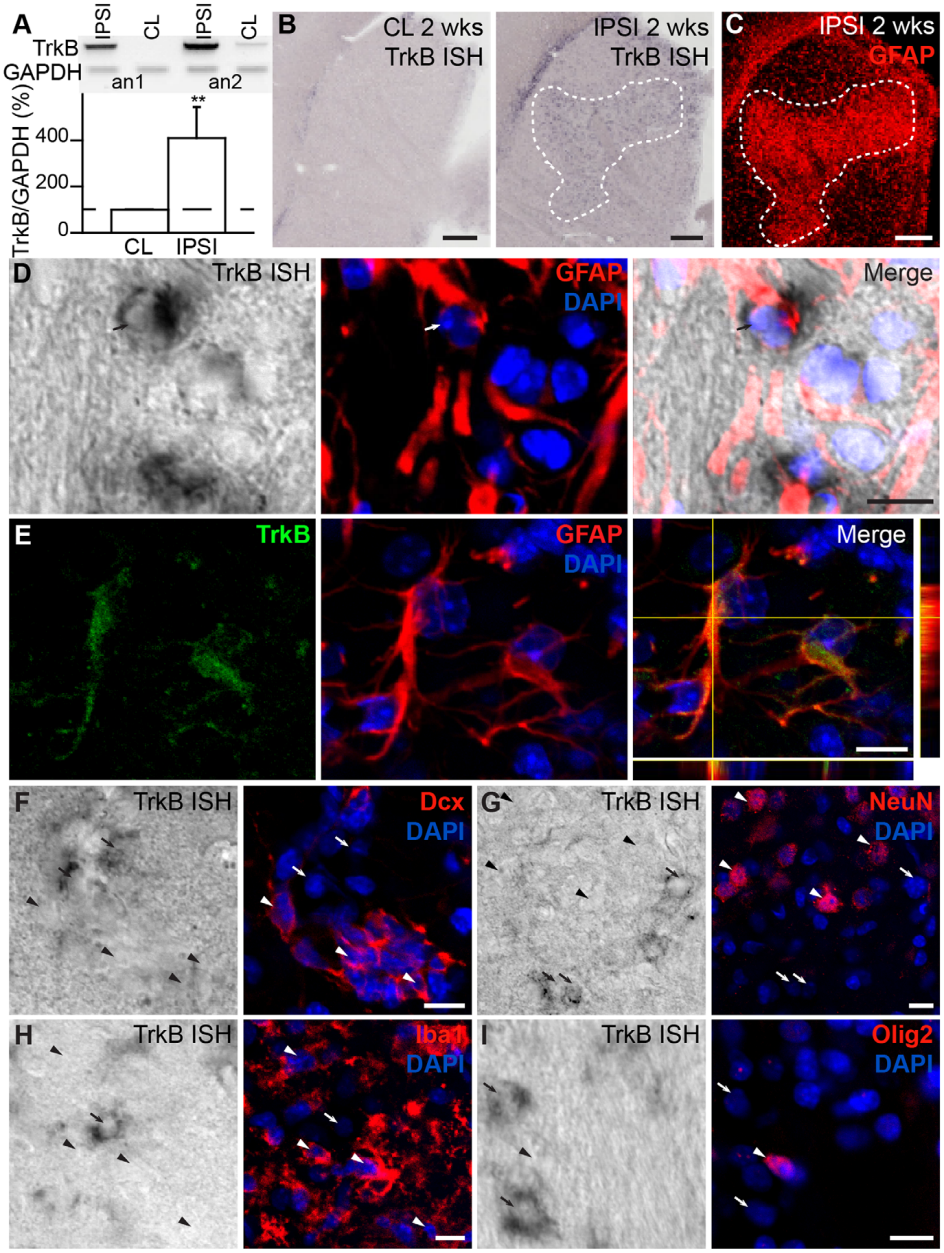
TrkB is expressed by reactive astrocytes in the ischemic striatum. ***A,*** The expression of TrkB in the ischemic striatum as assessed by PCR analysis one week after MCAo. Examples of contralateral (CL) and ipsilateral (IPSI) striata from 2 animals are shown. Low panel shows the quantification of TrkB expression in the ischemic striatum. ***B***, *In situ* hybridization revealed a massive upregulation of the TrkB receptor in the ipsilateral striatum (right), as opposed to virtually no TrkB expression in the contralateral side (left). The overexpression coincided with areas of astroglial accumulation (***C***). High magnification images revealed the clear apposition of the TrkB mRNA signal with GFAP immunolabeling (***D***, arrow). ***E***, Immunolabeling for TrkB and GFAP revealed that this neurotrophic receptor was expressed by astrocytes in the injured striatum. ***F–I,*** No TrkB mRNA was detected in Dcx^+^ neuroblasts (***F***), NeuN^+^ neurons (***G***), Iba1^+^ microglia (***H***)**,** or Olig2^+^ oligodendrocytes (***I***) (arrowheads; TrkB mRNA is indicated by arrows). The pattern of expression showed in these panels was observed 1 and 2 weeks post-MCAo. Scale bars: ***B***,***C***, 200 µm; ***D–I***, 10 µm (CL: contralateral; IPSI: ipsilateral; wk: week; ISH: *in situ* hybridization).

PCR analysis for TrkB (409.4±137.2% of change, *p*<0.01; n = 4 mice; Fig. 4A) and *in situ* hybridization with antisense riboprobe (Fig. 4B) revealed robust overexpression of the high-affinity receptor for BDNF throughout the ipsilateral striatum compared to the contralateral striatum. This pattern of expression was observed both 1 and 2 weeks post-MCAo and coincided with areas of massive astroglial accumulation (Fig. 4B,C). No signal was observed with sense riboprobe for TrkB in the contralateral and ipsilateral striata (Fig. S1). TrkB *in situ* hybridization combined with immunolabeling for cell type-specific markers confirmed the expression of TrkB by GFAP^+^ reactive astrocytes (Fig. 4D, arrow). Co-immunolabeling for GFAP and TrkB also showed that these proteins co-localized in the injured striatum (Fig. 4E). The expression of the TrkB receptor in astrocytes provided a clue concerning the BDNF immunolabeling results and suggested that the TrkB receptor in astrocytes “traps” extracellular BDNF released by endothelial and/or neuronal cells following ischemia. No TrkB mRNA or protein expression was observed in Dcx^+^ neuroblasts (Fig. 4F), NeuN^+^ neurons (Fig. 4G), Iba1^+^ microglia (Fig. 4H), or Olig2^+^ oligodendrocytes (Fig. 4I). The *in situ* hybridization and immunolabeling results for BDNF and its receptors in the ischemic striatum revealed a pattern that is strikingly similar to the expression pattern observed in the RMS [10]. As in the RMS, p75NTR^+^ neuroblasts in the ischemic striatum migrate along blood vessels expressing BDNF. Astrocytes that envelope blood vessels and contact neuroblasts likely trap extracellular BDNF through a high-affinity TrkB receptor. We thus investigated the dynamic properties of injury-induced neuroblast migration to determine whether BDNF acts directly on neuroblasts to guide their migration along the blood vessels in the ischemic striatum.

### Injury-induced Neuroblast Migration in the Striatum is Less Dynamic than Constitutive Migration in the RMS

**Figure 5:**
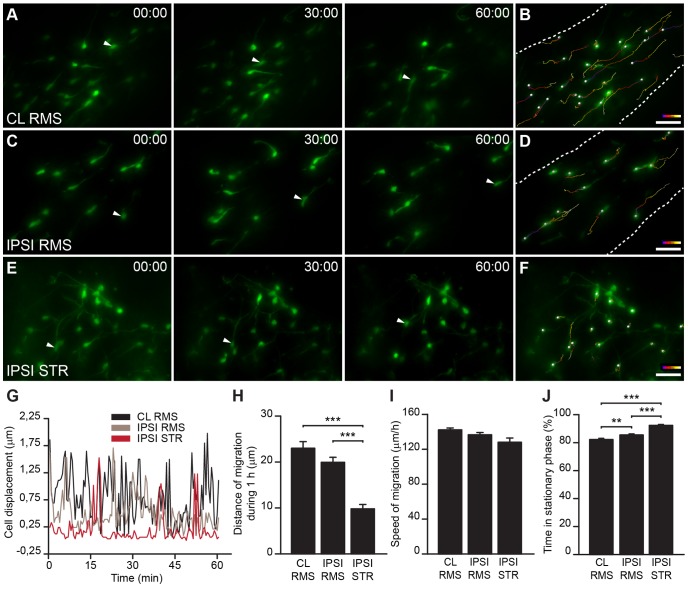
Dynamic properties of neuroblast migration in ischemic areas. ***A***–***F***, Timeline snapshots from real-time images of SVZ-derived precursor migration in acute brain slices of MCAo-challenged animals. Cells were labeled by injecting GFP-encoding retroviruses in the SVZ prior to inducing ischemia and were evaluated 2 to 3 weeks post-MCAo in the contralateral RMS (***A***,***B***), ipsilateral RMS (***C***,***D***), and ipsilateral striatum (***E,F***). Arrowheads indicate migrating cells. ***B***,***D***,***F,*** Migratory tracks of the cells migrating for 1 h in ***A,C,E***, respectively (color code: blue in the initial position; white in the final position). ***G***, Representative profile of cell displacement over time for individual neuroblasts migrating in the contralateral and ipsilateral RMS and the striatum. ***H*** **,** ***J,*** In the ipsilateral striatum, the recruited neuronal precursors displayed low migratory behavior, spending longer periods in the resting state (***J***), which resulted in shorter displacements per hour compared to the contralateral or ipsilateral RMS (***H***). ***I***, No differences in the speed of migration of neuroblasts migrating in the ischemic striatum and ipsilateral and contralateral RMS were observed. Scale bars: 20 µm (CL: contralateral; IPSI: ipsilateral; STR: striatum).

Despite a growing number of studies reporting that SVZ-derived progenitors migrate toward injured areas, the dynamic behavior of neuroblasts during such induced migration has not been fully explored. This issue has been approached by monitoring the migration of DiI-labeled cells, Dcx-GFP-expressing cells, and lentivirally-labeled cells in the striatum of organotypic slices from animals subjected to MCAo [7], [8], [36]. Time-lapse microscopy to monitor the displacement of recruited labeled cells in the striatum was performed, by acquiring multiple *z*-stack images every 15 min [8], [36] or 30 min [7]. While organotypic slices make it possible to follow cell migration for prolonged periods of times, culturing the slices may alter the migratory properties of the cells. In addition, acquisitions every 15 to 30 min make it difficult to detect and study some migratory properties such as the duration of the stationary and migratory phases, which can be as short as 4 to 10 min [10]. We thus acquired images every 30 s for 1–2 h in acute slices in order to monitor the patterns and dynamics of injury-induced migration in the striatum and compared them to normal RMS migration. We prepared slices 2 to 3 weeks post-ischemia and 3 to 4 weeks post-GFP-encoding lenti- or retrovirus injection into the SVZ and recorded the migration of de-routed neuroblasts in the ischemic striatum. We also recorded neuroblasts migration in the contralateral and ipsilateral RMS to determine whether ischemia-induced recruitment of neuronal precursors to the nearby striatum interferes with the normal dynamics of migration in the constitutive pathway. The analysis of neuroblasts migration in the RMS and the ischemic striatum clearly showed that neuroblasts migrated for shorter average distances per hour in the ipsilateral striatum compared to the contralateral (CL) or ipsilateral (IPSI) RMS (Fig. 5A–H *;* CL RMS: 34.48±2.18 µm, 121 cells, n = 16 video recordings obtained from 11 mice; IPSI RMS: 29.90±1.64 µm, 117 cells, n = 15 video recordings obtained from 11 mice; and STR: 14.70±1.43 µm, 74 cells, n = 37 video recordings obtained from 25 mice; *p*<0.001 with unpaired *t* test, for CL RMS vs. STR and IPSI RMS vs. STR). These results indicated that neuroblasts migrating in the ischemic striatum are less dynamic than those migrating in the normal environment. The difference in the total displacement distance of neuroblasts in the ischemic striatum compared to those in the RMS may be due to the changes in the speed of migration or differences in the duration of the migratory and stationary phases. In our analysis, we assessed the speed of migration solely during the migratory phases, as proposed earlier [10], [37]. Interestingly, there was no difference in the speed of migration between neuroblasts migrating in the ischemic striatum and in the contralateral or ipsilateral RMS (Fig. 5I; CL RMS: 213.3±3.26 µm/h, 121 cells, n = 16 video recordings obtained from 11 mice; IPSI RMS: 204.9±3.87 µm/h, 117 cells, n = 15 video recordings obtained from 11 mice; and STR: 192.0±7.32 µm/h, 74 cells, n = 37 video recordings obtained from 25 mice). The analysis of the duration of stationary phases revealed that neuroblasts spend significantly more time in the resting state when migrating in the ischemic striatum than in the contralateral or ipsilateral RMS (Fig. 5J; CL RMS: 82.18±0.88%, 121 cells, n = 16 video recordings obtained from 11 mice; IPSI RMS: 85.46±0.79%, 117 cells, n = 15 video recordings obtained from 11 mice; STR: 92.36±0.64%, 74 cells, n = 37 video recordings obtained from 25 mice; *p*<0.001 with unpaired *t* test, for CL RMS vs. STR, and for IPSI RMS vs. STR). Thus, fewer migratory periods of neuroblasts may account for the shorter displacement distances (track lengths) in the ischemic striatum (Fig. 5F). It also should be mentioned that most cells in the striatum were immotile during the 2 h recording period and were not taken into consideration. Representative traces of individual cells showed that the average distance that cells migrated in injured areas was shorter than in the RMS (Fig. 5G). In addition, while cell migration was highly directional in the RMS, once inside the injured striatal matrix, cells seemed to migrate randomly in different directions (in Fig. 5B,D,F, the range of colors in the tracks shows the initial (blue) and final (white) positions, and thus the direction of the movement). Interestingly, we also observed that neuroblasts in the RMS ipsilateral to the lesion have longer stationary periods than cells in the contralateral RMS (Fig. 5J; CL RMS: 82.18±0.88%, 121 cells, n = 16 video recordings obtained from 11 mice; IPSI RMS: 85.46±0.79%, 117 cells, n = 15 video recordings obtained from 11 mice; *p*<0.01 with unpaired *t* test). It is noteworthy that, unlike our previous studies on neuroblast migration in the naïve RMS [10], [28], in the present work we observed lower neuronal precursor motility and longer stationary phases in the contralateral RMS. This difference is likely due to the fact that we monitored cell migration in the RMS 3 to 4 weeks post-viral labeling of neuronal precursors in the SVZ, while in our previous studies we recorded cell migration in the RMS 4 to 7 days post-viral labeling in the SVZ [10], [28]. Thus, in the present work, we likely tracked a distinct population of neuroblasts that displayed lower motility and that remained in the RMS 3 to 4 weeks post-labeling in the SVZ.

**Figure 6:**
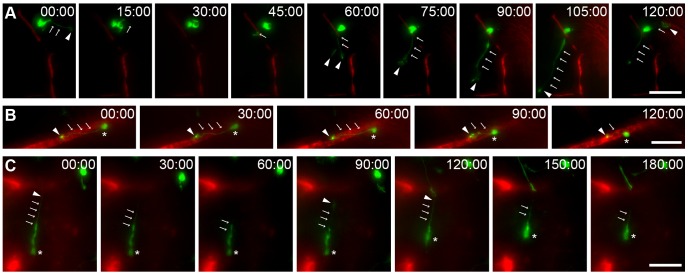
Dynamics of vasculature-mediated migration of neuroblasts in the ipsilateral striatum. *A–C* , Time-lapse videoimages of SVZ-derived neuroblasts migrating in acute brain slices prepared from MCAo-challenged animals. Cells were labeled by injecting GFP-encoding retroviruses in the SVZ (green) prior to inducing ischemia and were evaluated 2 to 3 weeks post-MCAo in the ipsilateral striatum. Blood vessels were visualized by dextran-Texas Red fluorescence (red). ***A***, In damaged areas, “resting” cells actively explored the surrounding environment by extending and retracting leading processes with a highly dynamic growth cone (arrows indicate the processes and arrowheads, the growth cone). ***B,*** An example of a GFP^+^ neuroblast migrating along a dextran-Texas Red-filled blood vessel. ***C,*** Time-lapse image showing that a GFP^+^ neuroblast located close to a blood vessel may extend a leading process toward another blood vessel and migrate in that direction by “jumping” from one blood vessel to another. In B and C, arrows indicate the processes, arrowheads the growth cone, and asterisks the cell soma. Scale bars: 20 µm.

In the ischemic striatum, “resting” cells with a migratory morphology often exhibited a highly exploratory behavior, constantly extending and retracting the leading process with a highly dynamic growth cone in different directions (Fig. 6A). The leading process stabilized on a number of occasions, and the cell soma was translocated in its direction, constituting a migratory event. Interestingly, most of the recruited neuroblasts migrated along blood vessels in the ischemic striatum (Fig. 6B and Movie S1). We observed two different types of migration. In the first case, neuroblasts migrated straight along the dextran-Texas Red-filled blood vessels (Fig. 6B). We also observed neuroblasts with their soma located close to one blood vessel and a leading process extended toward another blood vessel (Fig. 6C, arrowhead; see also Fig. 6A). In some cases, the growth cone at the end of the leading process stabilized, leading to the initiation of cell migration. These observations suggested that neuronal precursors may migrate in the post-stroke striatum by “jumping” from one blood vessel to another in addition to the “continuum” migration along individual blood vessels. These two types of migration modes may cooperate to increase the dispersal of recruited neuroblasts in the ischemic striatum.

### BDNF Promotes Injury-induced Migration in the Ischemic Striatum

**Figure 7:**
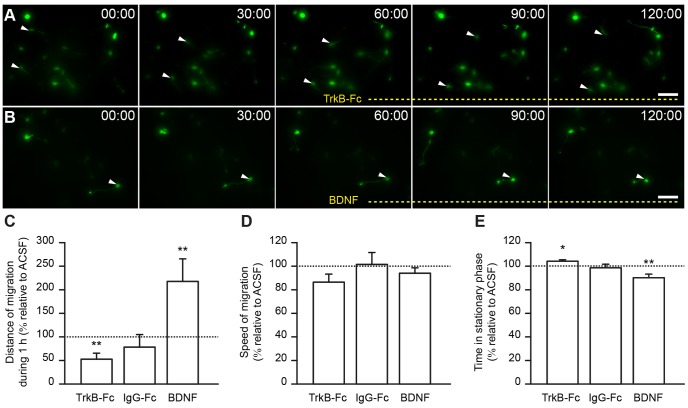
BDNF promotes neuroblast migration in ischemic areas. *A–B* , Time-lapse videoimages of neuroblasts migrating in the ischemic striatum under control conditions (0–60 min) and following the application (60–120 min) of TrkB-Fc (**A**) and BDNF (**B**). Bath-application of TrkB-Fc led to smaller displacement lengths due to an increase in the time spent by neuroblasts in the stationary period (***C,E***). On the other hand, the application of BDNF approximately doubled the total displacement per hour by reducing the time that cells spent in the stationary phase (***C,E***). Frequently, cells that were immotile during the first hour of recording initiated migration following BDNF perfusion (***B,*** arrowhead). ***D***, No differences in the speed of migration were observed following the application of TrkB-Fc, IgG-Fc, or BDNF. Scale bars: 20 µm.

To determine whether BDNF modulates the ischemia-induced migration of neuroblasts, we monitored neuroblast migration in the injured striatum of acute slices before and after the bath-application of BDNF or TrkB-Fc in order to scavenge endogenous BDNF. As a control we applied IgG-Fc. The pharmacological agents were applied during the second hour of recording and migration was compared between the first (control: ACSF) and second hour (pharmacological agent) of the recordings. Results are expressed as the percentage of the control (ACSF) for each group. TrkB-Fc, which scavenges extracellular BDNF, making it unavailable to the migrating cells, impaired neuroblast migration in the striatum (Fig. 7A and Movie S2). In control experiments, bath-application of IgG-Fc had no effect on migration. In contrast, the bath-application of recombinant BDNF fostered the migratory behavior of progenitor cells in the compromised striatum, often causing initiation of a migratory phase by previously resting cells (Fig. 7B and Movie S3). Quantification of neuroblast migration demonstrated that TrkB-Fc decreased the total length of neuroblast displacement per hour (52.77±12.63%, n = 15 cells, 6 video recordings obtained from 5 mice; *p*<0.01 with Student *t* test; Fig. 7C). In contrast, we observed a robust increase in total displacement following the application of BDNF (217.7±47.91%, n = 11 cells, 6 video recordings obtained from 4 mice; *p*<0.01 with Student *t* test; Fig. 7C). No effect on migration was observed in control experiments with IgG-Fc application (78.28±26.66%, n = 13 cells, 6 video recordings obtained from 4 mice; Fig. 7C). We observed no differences in the speed of migration following the application of TrkB-Fc, IgG-Fc, or BDNF (TrkB-Fc: 86.46±6.84%, n = 8 cells, 4 video recordings obtained from 4 mice; IgG-Fc: 101.5±10.15%, n = 6 cells, 4 video recordings obtained from 3 mice; BDNF: 94.02±4.61%, n = 8 cells, 6 video recordings obtained from 4 mice; Fig. 7D). Thus, whenever a migratory event was initiated, the cells displaced with the same speed during the migratory phase. However, TrkB-Fc increased the time spent in the stationary phase (104.2±1.23%, n = 15 cells, 6 video recordings obtained from 5 mice; *p*<0.05 with Student *t* test), whereas BDNF decreased the time spent by neuroblasts in the resting period (90.2±3%, n = 11 cells, 6 video recordings obtained from 4 mice; *p*<0.01 with Student *t* test) (Fig. 7E). No changes were observed following the bath-application of IgG-Fc (98.66±3.03%, n = 13 cells, 6 video recordings obtained from 4 mice; Fig. 7E). The changes in migration induced by BDNF and TrkB-Fc were thus due to changes in the time spent in the stationary phase (Fig. 7E). BDNF seemed to play a pivotal role in the switch from the stationary to the migratory phase by promoting the initiation of migration in cells navigating on the damaged striatum. These effects are similar to that of BDNF on migration in the RMS, where it promotes entry into the migratory phase [10].

## Discussion

The goal of the present study was to study the mechanisms of vasculature-mediated migration of neuronal precursors in the ischemic striatum. We demonstrated that neuroblasts de-routed to the injured striatum express p75NTR and migrate along striatal blood vessels, which synthesize BDNF in response to the injury. Trapping endogenous BDNF with TrkB-Fc impaired neuroblast migration in the acute slices prepared from the ischemic striatum, whereas recombinant BDNF triggered more migratory events by the de-routed cells. Interestingly, reactive astrocytes were closely associated with blood vessels and expressed TrkB, the high-affinity BDNF receptor. These glial cells may thus modulate the availability of BDNF for p75NTR-expressing neuroblasts by binding free extracellular BDNF. This would in turn modulate the migration of neuronal precursors as previously observed for the constitutive migration of neuroblasts in the RMS [10]. We also showed that neuroblasts migrating in the injured striatum display greater exploratory behavior and thus longer stationary periods than cells migrating in the RMS. Our findings showed that injury-induced migration is less dynamic than constitutive RMS migration, but that it is also controlled by BDNF signaling.

### Expression of BDNF and its Receptors after Ischemia

Unlike the intact striatum, the injured striatum contains considerable amounts of BDNF that is locally expressed by blood vessels and neuronal cells. Until recently, it was generally recognized that neurons are the major transient source of newly synthesized BDNF following strokes [12]–[14], [17], [38]. The expression of BDNF by surviving neurons in the degenerating ischemic cortex or striatum has been associated with an early autocrine neuroprotective role that counteracts cell death [39]–[41]. In recent years, other groups have shown that microglia transiently produce BDNF within 24 h of permanent focal [42] or multifocal cortical ischemia [18]. BDNF-immunolabeled ependymal and endothelial cells and astrocytes ensheathing vessels were also detected in injured areas [18]. However, since BDNF mRNA expression was not evaluated, the conclusions concerning the source of this secreted trophic factor based solely on immunoreactivity results must be treated with caution. In the present study, we used RT-PCR, *in situ* hybridization and immunohistochemistry to document the increased level of BDNF in the ischemic striatum and its expression by neurons and endothelial cells. Interestingly, while BDNF expression in NeuN^+^ striatal cells decayed from 1 to 2 weeks post-injury, BDNF expression in the striatal vasculature was maintained 2 weeks post-injury. This suggested that neuronal BDNF may be involved in early neuroprotective events, while endothelial BDNF, which persists for a longer time, promotes and maintains the migration of neuronal progenitors in an attempt to replace cells in the injured striatum.

We detected the BDNF receptor p75NTR, but not TrkB, in migrating neuroblasts, suggesting that the low-affinity p75NTR receptor is responsible for the stimulatory effect of BDNF on the neuroblast migration observed in the acute slices of injured striatum. In the SVZ, p75NTR is expressed by neuroblasts, type C cells, and a few astrocytes [10], [43]. Following an injury, some progenitors are mobilized from the SVZ and RMS to the striatum. However, the expression of the low-affinity BDNF receptor is maintained and is likely involved in cell navigation through the striatal matrix. In addition to neuroblasts, a few striatal neurons and astrocytes expressed p75NTR, while virtually no p75NTR was found in the contralateral striatum. Previous studies have shown that post-stroke neuronal upregulation of p75NTR takes place in a subpopulation of cholinergic striatal interneurons and likely prompts cells to undergo apoptosis [35], [44]. The functional role of p75NTR expression by astrocytes following ischemia remains, however, elusive. Studies on the intact cortex [45] and hippocampus [46] have suggested that p75NTR in astrocytes is involved in controlling the extracellular availability of BDNF.

TrkB, the high-affinity BDNF receptor, was expressed exclusively in activated astrocytes in the injured area. The expression of the full-length TrkB receptor in neuronal cells undergoes an acute transient increase following brain insults, but this lasts no longer than 24 h [47] or 48 h [48]. This suggests that TrkB plays a role in the early neuroprotective action of BDNF. The *in situ* probe and anti-TrkB antibody used in our study recognized both the full-length and truncated isoforms of TrkB receptor. However, it is widely accepted that the full-length receptor is typically abundant in neurons, whereas the truncated form is mainly found in glial cells [49]. This suggests that the TrkB receptor overexpressed by astrocytes that we observed in our study, at 1 and 2 weeks post-ischemia, may correspond to the truncated form of the receptor. In line with this, Wong et al. [31] also described a similar robust overexpression of truncated TrkB that persisted for at least 2 months after stab wound injury. Overexpression of TrkB by astrocytes may be involved in the regulation of neuroblast migration by controlling extracellular BDNF availability, as it is observed during constitutive migration of neuroblasts in the RMS [10].

### Molecular Mechanisms and Dynamics of SVZ Neuroblast Migration in the Ischemic Area

We explored the dynamic patterns of post-stroke migration by real-time video-imaging. It has been previously reported that neuroblasts in the ischemic striatum migrate at an average speed of 28.67±1.04 µm/h [8] or 33.1±1.6 µm/h [7]. In these studies, images were acquired every 15 min [8] or 30 min [7], and the calculation of the migratory speed encompassed both the resting and migratory phases (total displacement per time of recording). Since we acquired images every 30 s, we were able to separate the two phases and calculate the speed of cells migrating within the ischemic striatum or along the RMS solely during the migratory period. While our results revealed a marked difference in the duration and periodicity of the migratory and stationary phases in the ischemic striatum and RMS, we observed no changes in the speed of migration. Cells migrating in the striatum behaved as though they were actively screening the environment in different directions, with multiple extensions and retractions of the leading process and a highly dynamic growth cone. However, as soon as the migratory event was initiated, the neuroblasts migrated at the same speed as they do in the adult RMS. The increased exploratory behavior of the neuronal precursors in the injured striatum might have been caused by the “unusual” microenvironment, which does not fully recapitulate the cellular and molecular organization that facilitates neuroblast migration in the adult RMS. Moreover, invading cells face a detrimental environment rich in cell debris and death signals that might affect the migration of new neurons. These factors may account for the higher stationary periods observed during injury-induced migration compared to migration in the RMS.

Intraventricular infusion of BDNF or overexpression of the neurotrophin in the SVZ of adult rats leads to an increase in neuronal addition to the OB [50], [51] and aberrantly to the surrounding parenchyma, including striatum [51], [52]. These data were however contradicted by observations that intraventricular infusion of BDNF has no effect on neuron production from mouse SVZ, while decreased it in rats [53]. In addition, an increase in neuroblasts number within ischemic striatum was detected in transgenic BDNF+/− mice as compared to wild-type littermates [54]. These contrasting observations might be at least partially due to the diverse effect of BDNF on cell proliferation, differentiation, and survival, and the role of this trophic factor on SVZ neurogenesis is a subject of debate [50]–[57]. More consensuses exist regarding BDNF effect in the migration of neuronal precursors. Indeed, BDNF plays a pivotal role in neuronal migration during development [58]–[60] and in adulthood [10], [61]. Our group [10] and others [62], [63] have demonstrated a stimulatory effect of BDNF on migration of SVZ cells from the adult and postnatal brain. However, the difference exists in terms of implication of BDNF receptors in stimulating neuroblasts migration [10], [53], [62]. Chiaramello and colleagues have suggested that stimulatory effect of BDNF on neuroblasts migration occurs via TrkB receptors [62]. We [10] and others [53] have detected TrkB expression by SVZ-RMS astrocytes and p75NTR by migrating neuroblasts in mice [10] and rats [53]. We then showed that migration-promoting effect of BDNF occurs via p75NTR expressed by neuroblasts [10]. With regard to TrkB expression by astrocytes we suggested that this high-affinity receptor for BDNF may trap extracellular BDNF and thus modulate its availability for neuroblasts [10]. Interestingly, similar trapping effect was observed during migration of oligodendrocyte precursors where megalin expressing astrocytes sequester Shh to present it to oligodendrocytes precursors or to control the gradient of this molecule [64]. Using co-cultures of SVZ astrocytes and BDNF-GFP transfected endothelial cells we have previously shown that endothelial cell-derived BDNF is sequestered by TrkB-expressing astrocytes and that the trapping of this growth factor by glial cells is important for modulation of neuroblasts migration in the RMS [10]. We propose here that the same mechanisms might be also involved in the control of periodicity of migratory and stationary phases of migrating neuroblasts in the ischemic striatum.

### Conclusion

In the present study, we demonstrated that injury-induced migration also relies on a BDNF-dependent mechanism similar to constitutive neuronal migration in the adult SVZ-OB pathway. As in the adult RMS [10], 1) the migration of p75NTR-expressing neuroblasts occurred along the vasculature scaffold that synthesizes BDNF, 2) migration was fostered by BDNF, and 3) likely regulated by TrkB-expressing astrocytes that envelop the vessels and bind BDNF, thus controlling the extracellular levels of this trophic factor. The similarities between the mechanisms of constitutive and injury-induced vasculature-dependent migration of neuronal precursors suggests that some of the mechanisms controlling neuroblast migration in the adult RMS might be activated in the compromised striatum to recruit new cells. While weak, the spontaneous recruitment of neuronal precursors in the ischemic striatum suggests that there is potential for developing cell therapies that rely on promoting cell survival and/or injury-induced migration.

## Supporting Information

Figure S1 ***In situ*** **hybridization with control riboprobes.** *In situ* hybridization with sense BDNF (A) and TrkB (B) riboprobes of contralateral and ipsilateral striata, 1 week after MCAo.
(TIF)

Movie S1 **Vasculature-mediated migration in the ischemic striatum.** Time-lapse videoimaging of a GFP^+^ SVZ-derived neuroblast migrating along a dextran-Texas Red-filled blood vessel in the acute brain slices of the ischemic striatum.
(AVI)

Movie S2 **Trapping of endogenous BDNF with TrkB-Fc antibodies impairs neuroblast migration in the ischemic striatum.** Time-lapse videoimaging of GFP^+^ SVZ-derived neuroblasts migrating in the acute brain slices of the ischemic striatum. Application of TrkB-Fc (60–120 min), which scavenges the extracellular BDNF, leads to entrance into stationary phase thus hampering cell migration.
(AVI)

Movie S3 **BDNF stimulates neuroblast migration in the ischemic striatum.** Time-lapse videoimaging of GFP^+^ SVZ-derived neuroblasts migrating in the acute brain slices of the ischemic striatum. Application of BDNF (60–120 min) fosters the entrance of the cell into the migratory period.
(AVI)

## References

[1] Arvidsson A, Collin T, Kirik D, Kokaia Z, Lindvall O (2002) Neuronal replacement from endogenous precursors in the adult brain after stroke. Nat Med 8: 963–970. 12161747 10.1038/nm747

[2] Parent JM, Vexler ZS, Gong C, Derugin N, Ferriero DM (2002) Rat forebrain neurogenesis and striatal neuron replacement after focal stroke. Ann Neurol 52: 802–813. 12447935 10.1002/ana.10393

[3] Jin K, Sun Y, Xie L, Peel A, Mao XO, et al (2003) Directed migration of neuronal precursors into the ischemic cerebral cortex and striatum. Mol Cell Neurosci 24: 171–189. 14550778 10.1016/s1044-7431(03)00159-3

[4] Ohab JJ, Fleming S, Blesch A, Carmichael ST (2006) A neurovascular niche for neurogenesis after stroke. J Neurosci 26: 13007–13016. 17167090 10.1523/JNEUROSCI.4323-06.2006 PMC6674957

[5] Yamashita T, Ninomiya M, Hernandez Acosta P, Garcia-Verdugo JM, Sunabori T, et al (2006) Subventricular zone-derived neuroblasts migrate and differentiate into mature neurons in the post-stroke adult striatum. J Neurosci 26: 6627–6636. 16775151 10.1523/JNEUROSCI.0149-06.2006 PMC6674034

[6] Thored P, Wood J, Arvidsson A, Cammenga J, Kokaia Z, et al (2007) Long-term neuroblast migration along blood vessels in an area with transient angiogenesis and increased vascularization after stroke. Stroke 38: 3032–3039. 17901386 10.1161/STROKEAHA.107.488445

[7] Kojima T, Hirota Y, Ema M, Takahashi S, Miyoshi I, et al (2010) Subventricular zone-derived neural progenitor cells migrate along a blood vessel scaffold toward the post-stroke striatum. Stem Cells 28: 545–554. 20073084 10.1002/stem.306

[8] Zhang RL, Chopp M, Gregg SR, Toh Y, Roberts C, et al (2009) Patterns and dynamics of subventricular zone neuroblast migration in the ischemic striatum of the adult mouse. J Cereb Blood Flow Metab 29: 1240–1250. 19436318 10.1038/jcbfm.2009.55 PMC2741163

[9] Whitman MC, Fan W, Rela L, Rodriguez-Gil DJ, Greer CA (2009) Blood vessels form a migratory scaffold in the rostral migratory stream. J Comp Neurol 516: 94–104. 19575445 10.1002/cne.22093 PMC2746017

[10] Snapyan M, Lemasson M, Brill MS, Blais M, Massouh M, et al (2009) Vasculature guides migrating neuronal precursors in the adult mammalian forebrain via brain-derived neurotrophic factor signaling. J Neurosci 29: 4172–4188. 19339612 10.1523/JNEUROSCI.4956-08.2009 PMC6665362

[11] Bovetti S, Hsieh YC, Bovolin P, Perroteau I, Kazunori T, et al (2007) Blood vessels form a scaffold for neuroblast migration in the adult olfactory bulb. J Neurosci 27: 5976–5980. 17537968 10.1523/JNEUROSCI.0678-07.2007 PMC6672264

[12] Lindvall O, Ernfors P, Bengzon J, Kokaia Z, Smith ML, et al (1992) Differential regulation of mRNAs for nerve growth factor, brain-derived neurotrophic factor, and neurotrophin 3 in the adult rat brain following cerebral ischemia and hypoglycemic coma. Proc Natl Acad Sci U S A 89: 648–652. 1731336 10.1073/pnas.89.2.648 PMC48296

[13] Kokaia Z, Andsberg G, Yan Q, Lindvall O (1998) Rapid alterations of BDNF protein levels in the rat brain after focal ischemia: evidence for increased synthesis and anterograde axonal transport. Exp Neurol 154: 289–301. 9878168 10.1006/exnr.1998.6888

[14] Kokaia Z, Zhao Q, Kokaia M, Elmer E, Metsis M, et al (1995) Regulation of brain-derived neurotrophic factor gene expression after transient middle cerebral artery occlusion with and without brain damage. Exp Neurol 136: 73–88. 7589336 10.1006/exnr.1995.1085

[15] Schabitz WR, Steigleder T, Cooper-Kuhn CM, Schwab S, Sommer C, et al (2007) Intravenous brain-derived neurotrophic factor enhances poststroke sensorimotor recovery and stimulates neurogenesis. Stroke 38: 2165–2172. 17510456 10.1161/STROKEAHA.106.477331

[16] Keiner S, Witte OW, Redecker C (2009) Immunocytochemical detection of newly generated neurons in the perilesional area of cortical infarcts after intraventricular application of brain-derived neurotrophic factor. J Neuropathol Exp Neurol 68: 83–93. 19104443 10.1097/NEN.0b013e31819308e9

[17] Comelli MC, Guidolin D, Seren MS, Zanoni R, Canella R, et al (1993) Time course, localization and pharmacological modulation of immediate early inducible genes, brain-derived neurotrophic factor and trkB messenger RNAs in the rat brain following photochemical stroke. Neuroscience 55: 473–490. 8080474 10.1016/0306-4522(93)90517-j

[18] Béjot Y, Prigent-Tessier A, Cachia C, Giroud M, Mossiat C, et al (2011) Time-dependent contribution of non neuronal cells to BDNF production after ischemic stroke in rats. Neurochem Int 58: 102–111. 21074587 10.1016/j.neuint.2010.10.019

[19] Lalancette-Hébert M, Gowing G, Simard A, Weng YC, Kriz J (2007) Selective ablation of proliferating microglial cells exacerbates ischemic injury in the brain. J Neurosci 27: 2596–2605. 17344397 10.1523/JNEUROSCI.5360-06.2007 PMC6672496

[20] Engel O, Kolodziej S, Dirnagl U, Prinz V (2011) Modeling stroke in mice - middle cerebral artery occlusion with the filament model. J Vis Exp. (47), e2423, doi:10.3791/2423. 10.3791/2423 PMC3182649 21248698

[21] Thored P, Arvidsson A, Cacci E, Ahlenius H, Kallur T, et al (2006) Persistent production of neurons from adult brain stem cells during recovery after stroke. Stem Cells 24: 739–747. 16210404 10.1634/stemcells.2005-0281

[22] Zhang R, Zhang Z, Wang L, Wang Y, Gousev A, et al (2004) Activated neural stem cells contribute to stroke-induced neurogenesis and neuroblast migration toward the infarct boundary in adult rats. J Cereb Blood Flow Metab 24: 441–448. 15087713 10.1097/00004647-200404000-00009

[23] Peretto P, Merighi A, Fasolo A, Bonfanti L (1997) Glial tubes in the rostral migratory stream of the adult rat. Brain Res Bull 42: 9–21. 8978930 10.1016/s0361-9230(96)00116-5

[24] Kaneko N, Marin O, Koike M, Hirota Y, Uchiyama Y, et al (2010) New neurons clear the path of astrocytic processes for their rapid migration in the adult brain. Neuron 67: 213–223. 20670830 10.1016/j.neuron.2010.06.018 PMC4080818

[25] Mason HA, Ito S, Corfas G (2001) Extracellular signals that regulate the tangential migration of olfactory bulb neuronal precursors: inducers, inhibitors, and repellents. J Neurosci 21: 7654–7663. 11567055 10.1523/JNEUROSCI.21-19-07654.2001 PMC6762882

[26] García-Marqués J, De Carlos JA, Greer CA, López-Mascaraque L (2010) Different astroglia permissivity controls the migration of olfactory bulb interneuron precursors. Glia 58: 218–230. 19610095 10.1002/glia.20918 PMC3817351

[27] Bolteus AJ, Bordey A (2004) GABA release and uptake regulate neuronal precursor migration in the postnatal subventricular zone. J Neurosci 24: 7623–7631. 15342728 10.1523/JNEUROSCI.1999-04.2004 PMC6729616

[28] Bozoyan L, Khlghatyan J, Saghatelyan A (2012) Astrocytes control the development of the migration-promoting vasculature scaffold in the postnatal brain via VEGF signaling. J Neurosci 32: 1687–1704. 22302810 10.1523/JNEUROSCI.5531-11.2012 PMC6703370

[29] Hofer M, Pagliusi SR, Hohn A, Leibrock J, Barde YA (1990) Regional distribution of brain-derived neurotrophic factor mRNA in the adult mouse brain. Embo J 9: 2459–2464. 2369898 10.1002/j.1460-2075.1990.tb07423.x PMC552273

[30] Zermeno V, Espindola S, Mendoza E, Hernandez-Echeagaray E (2009) Differential expression of neurotrophins in postnatal C57BL/6 mice striatum. Int J Biol Sci 5: 118–127. 19173033 10.7150/ijbs.5.118 PMC2631221

[31] Wong JY, Liberatore GT, Donnan GA, Howells DW (1997) Expression of brain-derived neurotrophic factor and TrkB neurotrophin receptors after striatal injury in the mouse. Exp Neurol 148: 83–91. 9398452 10.1006/exnr.1997.6670

[32] Batchelor PE, Liberatore GT, Wong JY, Porritt MJ, Frerichs F, et al (1999) Activated macrophages and microglia induce dopaminergic sprouting in the injured striatum and express brain-derived neurotrophic factor and glial cell line-derived neurotrophic factor. J Neurosci 19: 1708–1716. 10024357 10.1523/JNEUROSCI.19-05-01708.1999 PMC6782182

[33] Lee TH, Abe K, Kogure K, Itoyama Y (1995) Expressions of nerve growth factor and p75 low affinity receptor after transient forebrain ischemia in gerbil hippocampal CA1 neurons. J Neurosci Res 41: 684–695. 7563249 10.1002/jnr.490410515

[34] Oderfeld-Nowak B, Orzylowska-Sliwinska O, Soltys Z, Zaremba M, Januszewski S, et al (2003) Concomitant up-regulation of astroglial high and low affinity nerve growth factor receptors in the CA1 hippocampal area following global transient cerebral ischemia in rat. Neuroscience 120: 31–40. 12849738 10.1016/s0306-4522(03)00289-6

[35] Andsberg G, Kokaia Z, Lindvall O (2001) Upregulation of p75 neurotrophin receptor after stroke in mice does not contribute to differential vulnerability of striatal neurons. Exp Neurol 169: 351–363. 11358448 10.1006/exnr.2001.7646

[36] Zhang RL, LeTourneau Y, Gregg SR, Wang Y, Toh Y, et al (2007) Neuroblast division during migration toward the ischemic striatum: a study of dynamic migratory and proliferative characteristics of neuroblasts from the subventricular zone. J Neurosci 27: 3157–3162. 17376977 10.1523/JNEUROSCI.4969-06.2007 PMC6672487

[37] Bortone D, Polleux F (2009) KCC2 expression promotes the termination of cortical interneuron migration in a voltage-sensitive calcium-dependent manner. Neuron 62: 53–71. 19376067 10.1016/j.neuron.2009.01.034 PMC3314167

[38] Takeda A, Onodera H, Sugimoto A, Kogure K, Obinata M, et al (1993) Coordinated expression of messenger RNAs for nerve growth factor, brain-derived neurotrophic factor and neurotrophin-3 in the rat hippocampus following transient forebrain ischemia. Neuroscience 55: 23–31. 8350988 10.1016/0306-4522(93)90451-k

[39] Lindvall O, Kokaia Z, Bengzon J, Elmer E, Kokaia M (1994) Neurotrophins and brain insults. Trends Neurosci 17: 490–496. 7531892 10.1016/0166-2236(94)90139-2

[40] Saarelainen T, Lukkarinen JA, Koponen S, Grohn OH, Jolkkonen J, et al (2000) Transgenic mice overexpressing truncated trkB neurotrophin receptors in neurons show increased susceptibility to cortical injury after focal cerebral ischemia. Mol Cell Neurosci 16: 87–96. 10924253 10.1006/mcne.2000.0863

[41] Larsson E, Nanobashvili A, Kokaia Z, Lindvall O (1999) Evidence for neuroprotective effects of endogenous brain-derived neurotrophic factor after global forebrain ischemia in rats. J Cereb Blood Flow Metab 19: 1220–1228. 10566968 10.1097/00004647-199911000-00006

[42] Madinier A, Bertrand N, Mossiat C, Prigent-Tessier A, Beley A, et al (2009) Microglial involvement in neuroplastic changes following focal brain ischemia in rats. PLoS One 4: e8101. 19956568 10.1371/journal.pone.0008101 PMC2779656

[43] Young KM, Merson TD, Sotthibundhu A, Coulson EJ, Bartlett PF (2007) p75 neurotrophin receptor expression defines a population of BDNF-responsive neurogenic precursor cells. J Neurosci 27: 5146–5155. 17494700 10.1523/JNEUROSCI.0654-07.2007 PMC6672366

[44] Angelo MF, Aviles-Reyes RX, Villarreal A, Barker P, Reines AG, et al (2009) p75 NTR expression is induced in isolated neurons of the penumbra after ischemia by cortical devascularization. J Neurosci Res 87: 1892–1903. 19156869 10.1002/jnr.21993

[45] Bergami M, Santi S, Formaggio E, Cagnoli C, Verderio C, et al (2008) Uptake and recycling of pro-BDNF for transmitter-induced secretion by cortical astrocytes. J Cell Biol 183: 213–221. 18852301 10.1083/jcb.200806137 PMC2568011

[46] Dougherty KD, Milner TA (1999) p75NTR immunoreactivity in the rat dentate gyrus is mostly within presynaptic profiles but is also found in some astrocytic and postsynaptic profiles. J Comp Neurol 407: 77–91. 10213189 10.1002/(sici)1096-9861(19990428)407:1<77::aid-cne6>3.0.co;2-s

[47] Merlio JP, Ernfors P, Kokaia Z, Middlemas DS, Bengzon J, et al (1993) Increased production of the TrkB protein tyrosine kinase receptor after brain insults. Neuron 10: 151–164. 8439408 10.1016/0896-6273(93)90307-d

[48] Narumiya S, Ohno M, Tanaka N, Yamano T, Shimada M (1998) Enhanced expression of full-length TrkB receptors in young rat brain with hypoxic/ischemic injury. Brain Res 797: 278–286. 9666147 10.1016/s0006-8993(98)00385-0

[49] Reichardt LF (2003) Neurobiology: signals that make waves. Nature 426: 25–26. 14603298 10.1038/426025a

[50] Zigova T, Pencea V, Wiegand SJ, Luskin MB (1998) Intraventricular administration of BDNF increases the number of newly generated neurons in the adult olfactory bulb. Mol Cell Neurosci 11: 234–245. 9675054 10.1006/mcne.1998.0684

[51] Benraiss A, Chmielnicki E, Lerner K, Roh D, Goldman SA (2001) Adenoviral brain-derived neurotrophic factor induces both neostriatal and olfactory neuronal recruitment from endogenous progenitor cells in the adult forebrain. J Neurosci 21: 6718–6731. 11517261 10.1523/JNEUROSCI.21-17-06718.2001 PMC6763117

[52] Pencea V, Bingaman KD, Wiegand SJ, Luskin MB (2001) Infusion of brain-derived neurotrophic factor into the lateral ventricle of the adult rat leads to new neurons in the parenchyma of the striatum, septum, thalamus, and hypothalamus. J Neurosci 21: 6706–6717. 11517260 10.1523/JNEUROSCI.21-17-06706.2001 PMC6763082

[53] Galvão RP, Garcia-Verdugo JM, Alvarez-Buylla A (2008) Brain-derived neurotrophic factor signaling does not stimulate subventricular zone neurogenesis in adult mice and rats. J Neurosci 28: 13368–13383. 19074010 10.1523/JNEUROSCI.2918-08.2008 PMC2659623

[54] Nygren J, Kokaia M, Wieloch T (2006) Decreased expression of brain-derived neurotrophic factor in BDNF(+/−) mice is associated with enhanced recovery of motor performance and increased neuroblast number following experimental stroke. J Neurosci Res 84: 626–631. 16770774 10.1002/jnr.20956

[55] Chmielnicki E, Benraiss A, Economides AN, Goldman SA (2004) Adenovirally expressed noggin and brain-derived neurotrophic factor cooperate to induce new medium spiny neurons from resident progenitor cells in the adult striatal ventricular zone. J Neurosci 24: 2133–2142. 14999064 10.1523/JNEUROSCI.1554-03.2004 PMC6730416

[56] Cheng A, Wang S, Cai J, Rao MS, Mattson MP (2003) Nitric oxide acts in a positive feedback loop with BDNF to regulate neural progenitor cell proliferation and differentiation in the mammalian brain. Dev Biol 258: 319–333. 12798291 10.1016/s0012-1606(03)00120-9

[57] Bath KG, Mandairon N, Jing D, Rajagopal R, Kapoor R, et al (2008) Variant brain-derived neurotrophic factor (Val66Met) alters adult olfactory bulb neurogenesis and spontaneous olfactory discrimination. J Neurosci 28: 2383–2393. 18322085 10.1523/JNEUROSCI.4387-07.2008 PMC2679965

[58] Ahmed S, Reynolds BA, Weiss S (1995) BDNF enhances the differentiation but not the survival of CNS stem cell-derived neuronal precursors. J Neurosci 15: 5765–5778. 7643217 10.1523/JNEUROSCI.15-08-05765.1995 PMC6577638

[59] Borghesani PR, Peyrin JM, Klein R, Rubin J, Carter AR, et al (2002) BDNF stimulates migration of cerebellar granule cells. Development 129: 1435–1442. 11880352 10.1242/dev.129.6.1435

[60] Polleux F, Whitford KL, Dijkhuizen PA, Vitalis T, Ghosh A (2002) Control of cortical interneuron migration by neurotrophins and PI3-kinase signaling. Development 129: 3147–3160. 12070090 10.1242/dev.129.13.3147

[61] Marin O, Rubenstein JL (2003) Cell migration in the forebrain. Annu Rev Neurosci 26: 441–483. 12626695 10.1146/annurev.neuro.26.041002.131058

[62] Bath KG, Akins MR, Lee FS (2012) BDNF control of adult SVZ neurogenesis. Dev Psychobiol. In press. 10.1002/dev.20546 PMC3139728 21432850

[63] Chiaramello S, Dalmasso G, Bezin L, Marcel D, Jourdan F, et al (2007) BDNF/TrkB interaction regulates migration of SVZ precursor cells via PI3-K and MAP-K signalling pathways. Eur J Neurosci 26: 1780–1790. 17883412 10.1111/j.1460-9568.2007.05818.x

[64] Louissaint A Jr, Rao S, Leventhal C, Goldman SA (2002) Coordinated interaction of neurogenesis and angiogenesis in the adult songbird brain. Neuron 34: 945–960. 12086642 10.1016/s0896-6273(02)00722-5

[65] Ortega MC, Cases O, Merchan P, Kozyraki R, Clemente D, et al (2012) Megalin mediates the influence of sonic hedgehog on oligodendrocyte precursor cell migration and proliferation during development. Glia 60: 851–866. 22354480 10.1002/glia.22316

