# Supplementary figures and images for: Brain-Derived Neurotrophic Factor Promotes Vasculature-Associated Migration of Neuronal Precursors toward the Ischemic Striatum
Source: PLoS One. 2013 Jan 29;8(1):e55039. doi: 10.1371/journal.pone.0055039 (PMC3558494; doi:10.1371/journal.pone.0055039)

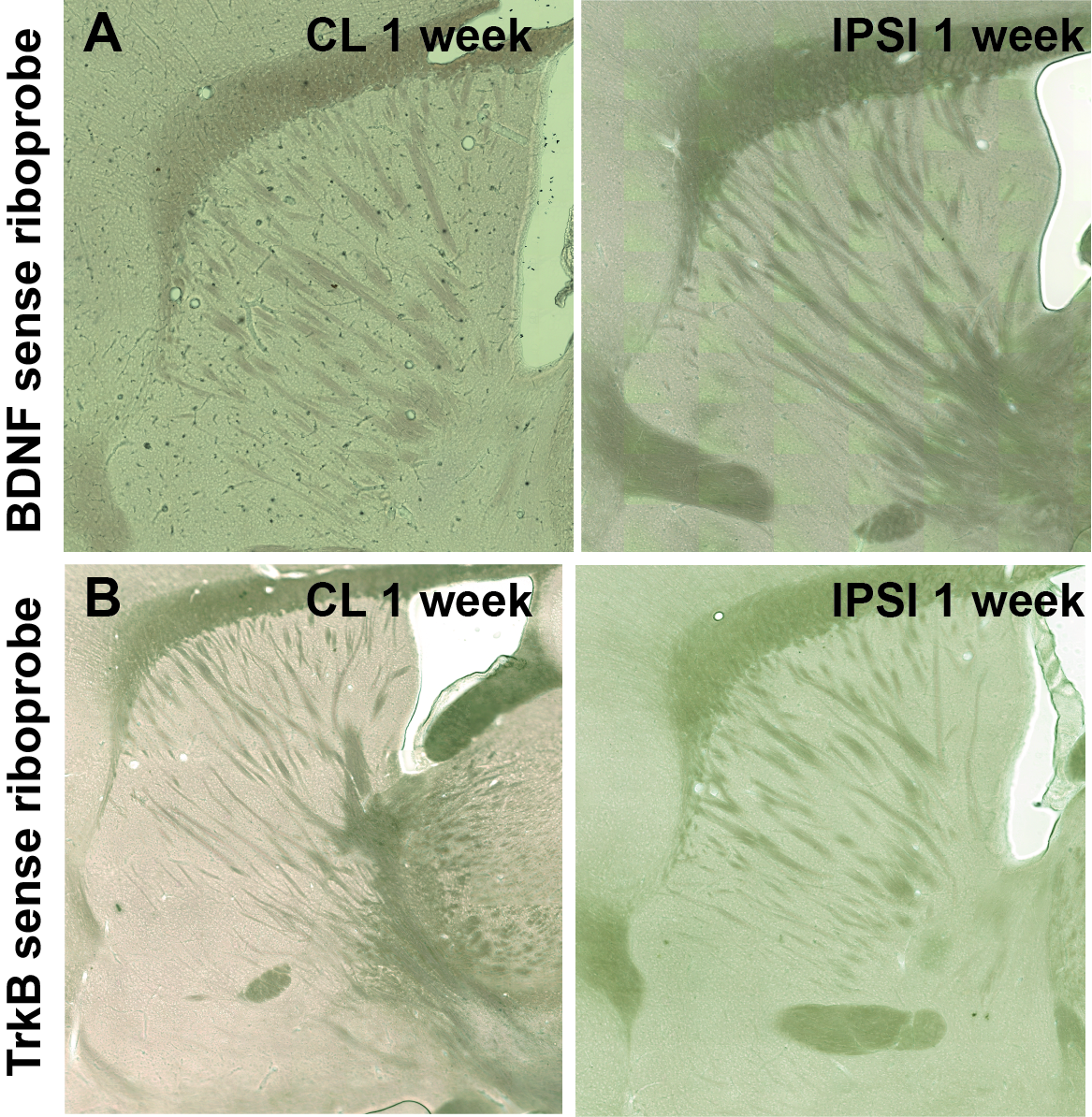

Supplement: Figure S1 — In situ hybridization with control riboprobes. In situ hybridization with sense BDNF (A) and TrkB (B) riboprobes of contralateral and ipsilateral striata, 1 week after MCAo. (TIF) [file pone.0055039.s001.tif]
